# Medication‐related osteonecrosis of the jaws: a single centre, Far North Queensland case series

**DOI:** 10.1111/adj.12905

**Published:** 2022-03-18

**Authors:** S Smith, B Finn, AN Goss

**Affiliations:** ^1^ Cairns Dental Anaesthetic Centre Cairns Queensland Australia; ^2^ Oral and Maxillofacial Surgeon Cairns Queensland Australia; ^3^ Emeritus Professor of Oral & Maxillofacial Surgery The University of Adelaide Adelaide South Australia Australia

**Keywords:** bisphosphonates, demographics, denosumab, MRONJ, treatment

## Abstract

Medication‐related osteonecrosis of the jaws (MRONJ) is a painful debilitating condition which is considered rare in the medical literature available to prescribers. Dentists, however, are likely to trigger this condition through extractions and implants. Anecdotally MRONJ appears more common than first thought. This paper presents 13 cases of MRONJ diagnosed and treated by a single oral and maxillofacial surgeon based in Cairns, Far North Queensland, in a 2‐year period. The management of these cases is presented. The two cases where MRONJ resulted in the loss of dental implants are highlighted. © 2022 Australian Dental Association

**Abbreviations and acronyms**
CTX: serum beta cross laps test
FNQ: Far North Queensland
MRONJ: Medication‐related osteonecrosis of the jaws
OMS: oral and maxillofacial surgery

## INTRODUCTION

Medication‐related osteonecrosis of the jaws (MRONJ) is defined as an area of exposed jaw bone of greater than 8 weeks duration in patients on antiresorptive or antiangiogenic medication.^1^ It is staged from ‘At risk’ through to Stage III for extensive necrosis. One of the key issues is that the medications are prescribed by medical practitioners for osteoporosis or bone cancer and their literature is that MRONJ is rare, at a rate of 1 in 1000–10 000.^2^, ^3^ On the other hand the condition is triggered by dental procedures and MRONJ management, particularly for severe cases, is by oral and maxillofacial surgeons. The few independent studies show that MRONJ is not rare. In one consecutive series of patients on oral bisphosphonates for osteoporosis four cases of MRONJ were found in 950 patients having dental extractions. This is 1 in 238. When this group was further stratified, via a fasted serum beta cross laps test (CTX), it was found that in the patients with a bone turnover of less than 150 pg/mL then all 4 cases were in that group of 180 or 1 in 45.^4^ In a separate but similar study of patients on denosumab for osteoporosis, 10 patients out of 484 patients, having dental extractions, developed MRONJ or 1 in 48.^5^ These higher rates of MRONJ, in patients treated with antiresorptives for osteoporosis where the bone turnover is low, are similar to those seen in patients being treated for bone cancer.^6^ For oncologic purposes higher doses of stronger antiresorptives are used for longer periods thus with greater suppression of bone turnover.

To further document the number and type of MRONJ cases presenting to an oral and maxillofacial surgeon’s private rooms in Far North Queensland, we examined the case records for the 2‐year period 2019 to 2020.

## METHOD AND MATERIALS

The records of an OMS private practice in Cairns, Far North Queensland, for the period January 2019 to December 2020 were reviewed and all new patients presenting in that time period diagnosed with MRONJ were identified. The full clinical and radiographic records were reviewed and the following data recorded; age at diagnosis; gender; medical status; bone condition; type and duration of antiresorptives; trigger for MRONJ; stage; results of treatment and CTX data. The data were recorded following the standard classifications used for MRONJ studies.^4^, ^5^ Thus, for example the medical status of patients was recorded as ‘fit and well’ for patients not having any medical conditions apart from their bone condition. ‘Stable medicated’ for patients who were undergoing medical treatment for conditions such as heart and respiratory diseases which did not affect their bones. ‘Immunocompromised’ related to patients with Type I diabetes, chronic steroid use or undergoing treatment for malignancy.

All patients, in addition to standard OMS history, examination and radiographs, had a fasted CTX taken on presentation. The referring practitioner was also asked if they had taken any CTX tests. All 13 patients gave consent to their histories to be included in a de‐identified manner.

## RESULTS

All the patients were referred to the OMS with MRONJ by their medical or dental practitioner thus the triggering dental treatment of extractions or irritation of tori by dentures had occurred elsewhere. The two patients with MRONJ associated with dental implants had had their implants initially placed by the OMS, with their subsequent restoration and follow‐up performed by their general dentist. When they developed problems, they were referred back to the OMS.

The three patients with cancer were all females with breast cancer, two had established metastasis and one was on antiresorptives to minimize the risk of metastasis. All were immunocompromised.

The two patients with MRONJ on palatal tori had a spontaneous onset, probably secondary to masticatory trauma. One of these patients was immunocompromised.

Treatment followed the principles set out in the recent publication in the ADA Newsletter.^7^ Namely, chlorhexidine‐based mouth rinses, antibiotics if soft tissue infection and sequestrectomy of dead bone. Alternative management of their underlying bone condition was held with the patient’s treating medical practitioner. The results are presented in Table 1.

**Table 1:** Summary of the 13 cases*

Age	72 (59–87)
Gender	
Male	2
Female	11
Medical status	
I (Fit and well)	1
II (Stable, medicated)	7
III (Immunocompromised)	5
Condition	
Osteoporosis	10
Bone Cancer	3
Medication	
Bisphosphonate	
IV	1
Oral	0
Denosumab	8
Denosumab with prior bisphosphonate	4
Duration of antiresorptives	
Less than 4 years	2
Greater than 4 years	11
Trigger	
Extraction	9
Implants	2
Tori	2
Stage	
I	0
II	10
III	3
Status	
Healed	9
Not healed	1
Not known	3
CTX Levels	
At onset	
Below 150 pg/mL	11
Above 150 pg/mL	1
3/12 After onset	
Above 150 pg/mL 3/12	1

* The 13 patients gave consent for their histories to be included in a de‐identified manner.

## DISCUSSION

This case series shows that MRONJ is a common presentation to a single part time private practice for an oral and maxillofacial surgeon in Cairns, Far North Queensland. It does not include the patients with MRONJ seen through the same surgeon’s Cairns Base Hospital public outpatient clinics. In that public environment more cases of bone malignancy MRONJ were seen, secondary to oncological doses of antiresorptive and antiangiogenic drugs.

Far North Queensland is a vast tropical area of over 381 000 square kilometres with a sparse population of 290 000. There is a predominance of males (53%) and a relatively young median age of 31 years. Only approximately 30 000 of this population are over 55‐year‐old females which is the most likely age for osteoporosis. There is also a relatively higher population of itinerant workers and holiday makers. Thus, there is a different demographic to the more densely populated urban areas in the southern states where people are older, average 37 years, and 52% are females.^8^ Oral and maxillofacial surgery services are limited in FNQ, with only one resident OMS in Cairns providing both public and private services.

The overall presentation as set out Table 1 is typical of other series, over 70, female, on denosumab for osteoporosis for more 4 years, triggered by dental extraction, with their CTX less than 150 pg/mL and MRONJ at Stage 2.^4^, ^5^ The different FNQ demographic, however, does show a similar MRONJ population to southern metropolitan Australia. Treatment followed and standard approach of patient and medical prescriber education of the nature of the condition. Pain was treated with analgesics, a short course of antibiotic for soft tissue infection and chlorhexidine mouth rinses. Radiographs were taken to demonstrate the extent. Any dead bone was removed under local anaesthesia and the soft tissues closed.^7^ Nine of the 13 healed within a few months, one continued to heal and three who were unknown. These three all came from a considerable distance away from Cairns so follow‐up was with their local medical and dental practitioners.

One patient, who was the oldest at 87, was medically compromised and had a simple extraction of tooth 32, had initial healing but represented 2 months later with pain. On review, it was found that the patient’s medical practitioner had insisted that they have the next dose of denosumab at 10 days of post‐extraction. This was before primary bone healing had occurred so resulted in MRONJ.^9^ Similar cases of premature recommencement of denosumab resulting in MRONJ have been reported.^5^

**Fig. 1:**
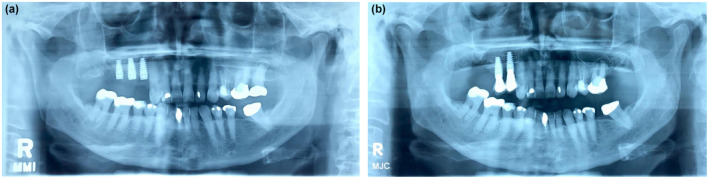
(a) Case 1→11/8/2015: At insertion of implants. (b) Case 1→11/8/2020: Stage III MRONJ at 16, and loss of attachment 15, 14. [Colour figure can be viewed at wileyonlinelibrary.com]

The two patients with implants which failed are different but show important characteristics. If their medical practitioner had enquired about their dental health, they could both report it was excellent. Thus, there would have been no medical contraindication to changing their oral bisphosphonate to denosumab. One aged 83, female was on Actonel for osteoporosis at the time of implant placement. She had implants placed at 14, 15 and 16 with a sinus lift with mixed Bio Oss and mandibular bone graft, with good primary stability (Fig. 1a). She was changed to denosumab after 4 years and after the third dose, MRONJ developed. Non‐surgical treatment was initially commenced and after a further 4 months the 16 spontaneously exfoliated and the 15 was loose and removed. Denosumab was ceased, the implants and sequestra removed and the Stage III MRONJ slowly resolved (Fig. 1b).

**Fig. 2:**
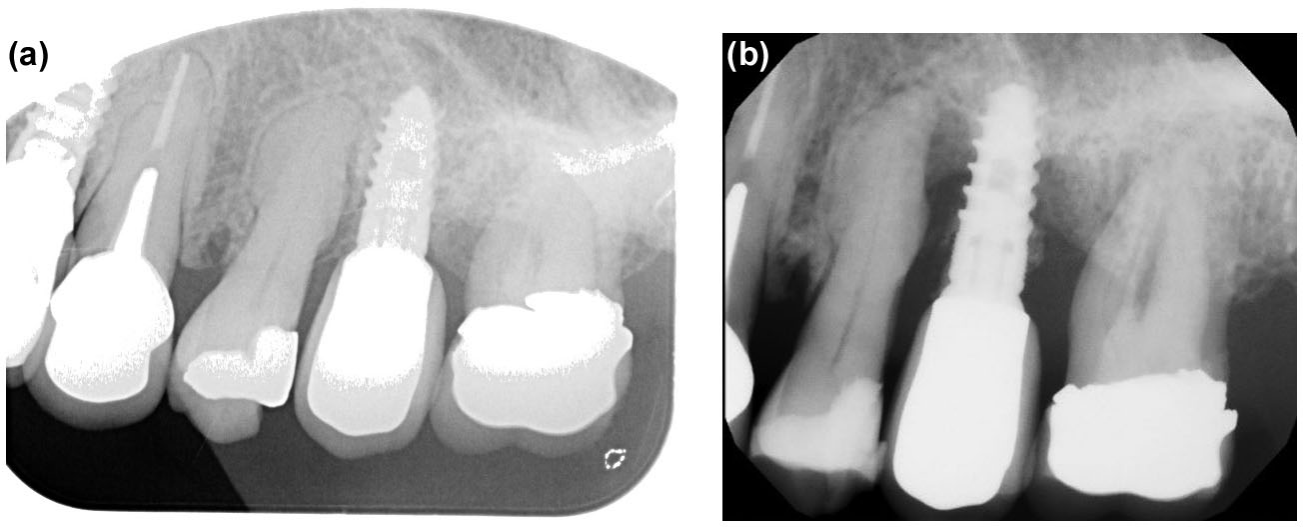
(a) Case 2→17/7/2017: Implants 4 years after insertion. (b) Case 2→5/11/2020: Periapical radiograph showing bone loss around implant at 25.

The second patient was a 67‐year‐old female who was initially on Actonel for osteoporosis in 2007 and changed to denosumab in 2013. Dental implants were placed in 2015 at sites 1, 15 and 25 with a bone graft from the mandible and Bio Oss and a Bio Gide membrane (Fig. 2a). After 5 years, in 2020 there was bleeding and odour was associated with the 25 implant. This was initially diagnosed as advanced periimplantitis but subsequently diagnosed as MRONJ. (Fig. 2b). The crown was removed and a healing abutment was placed. The patient was initially taken off denosumab but had developed breast cancer which was not suitable for oestrogen therapy or teriparatide. Accordingly, she was returned to Actonel. Close periodontal monitoring is in place and time will tell whether it resolves or the implant is exfoliated. The remaining implants show no evidence of loss of integration.

These two cases show the complexity of management of implant osseointegration in the presence of antiresorptives. Although the risk of implant loss is low, less than 5%, it is a very real and expensive loss to the patient. Similar findings have been reported for patients with dental implants who were on oral bisphosphonates.^10^

There were two cases where MRONJ occurred secondary to mucosal trauma to palatal tori. Both were on denosumab for osteoporosis. Again tori as a risk factor is not well recognized medically and often are overlooked dentally. One case was treated non surgically and the other required surgical removal of the tori. MRONJ from tori, secondary to minor trauma, are more common presentations in oncology patients, where there is a higher dose.

At all stages, there needs to be close communication between the prescriber of the antiresorptives for important medical conditions such as osteoporosis and bone cancers on the one hand and the dental profession on the other. Both need to be aware of the benefits and risks of treatment and provide informed consent advice to the patient.

Minimization of the risk of MRONJ is best by avoiding the known triggers. Timing is important particularly for patients on denosumab for osteoporosis and can be guided by measuring the bone turnover by CTX testing. This simple laboratory test can be ordered by any registered dentist via a pathology laboratory with the requirement that the form states the reason for the test, that is ‘patient on an antiresorptive medication for osteoporosis requiring an extraction.’ There has been some controversy and misunderstanding about this test^11^ but it is a useful guide. It needs to be fasted and unexercised, performed close to the extraction and knowing that it gives an estimation of whether the patient is in the ‘risk zone’ rather than the certainty that the patient will develop MRONJ.^12^, ^13^

Careful attention also needs to be considered for the ongoing management of osteoporosis. Once the patient hears that their painful jaw was caused by their medication, they often wish to stop taking it. With that action, however, they risk low‐impact fractures or particularly with denosumab of vertebral collapse. These patients need to be on some type of antiresorptive and the opinion of a specialist endocrinologist, with an interest in bone diseases, should be sought.
